# Supplementary material for: Genome-Wide Identification of the Dof Gene Family and Functional Analysis of PeSCAP1 in Regulating Guard Cell Maturation in Populus euphratica
Source: Int J Mol Sci. 2025 Apr 17;26(8):3798. doi: 10.3390/ijms26083798 (PMC12028277; doi:10.3390/ijms26083798)
Supplement: Supplementary file 1 [file ijms-26-03798-s001.zip › ijms-3510412-supplementary.pdf]

Supporting Information

The following Supporting Information is available for this article:

Figure S1. Confirmation of identified PeDofs using HMMER online software;

Figure S2. Prediction of conserved motifs in PeDof proteins;

Figure S3. Neighbor-joining (NJ) tree of *P. euphratica* and *A. thaliana*;

Figure S4. Sequencing analysis of *AtSCAP1* in the *atscap1-3* mutant;

Figure S5. The relative expression levels of genes related to stomatal maturation.

Table S1 Primers used in this study.

Sequence Number	Query Name	Hits Found	Status	Identifier	Description
1	PeuTF05G01648.1	1	DONE	zf-Dof	Dof domain, zinc finger
2	PeuTF02G00480.1	1	DONE	zf-Dof	Dof domain, zinc finger
3	PeuTF11G00428.1	1	DONE	zf-Dof	Dof domain, zinc finger
4	PeuTF16G00617.1	1	DONE	zf-Dof	Dof domain, zinc finger
5	PeuTF03G00284.1	1	DONE	zf-Dof	Dof domain, zinc finger
6	PeuTF14G01584.1	1	DONE	zf-Dof	Dof domain, zinc finger
7	PeuTF15G00470.1	1	DONE	zf-Dof	Dof domain, zinc finger
8	PeuTF07G00397.1	1	DONE	zf-Dof	Dof domain, zinc finger
9	PeuTF12G00528.1	1	DONE	zf-Dof	Dof domain, zinc finger
10	PeuTF05G01114.1	1	DONE	zf-Dof	Dof domain, zinc finger
11	PeuTF11G00355.1	1	DONE	zf-Dof	Dof domain, zinc finger
12	PeuTF07G00415.1	1	DONE	zf-Dof	Dof domain, zinc finger
13	PeuTF11G00419.1	1	DONE	zf-Dof	Dof domain, zinc finger
14	PeuTF05G01128.1	1	DONE	zf-Dof	Dof domain, zinc finger
15	PeuTF19G00358.1	1	DONE	zf-Dof	Dof domain, zinc finger
16	PeuTF11G00528.1	1	DONE	zf-Dof	Dof domain, zinc finger
17	PeuTF04G00386.1	1	DONE	zf-Dof	Dof domain, zinc finger
18	PeuTF06G01955.1	1	DONE	zf-Dof	Dof domain, zinc finger
19	PeuTF15G00102.1	1	DONE	zf-Dof	Dof domain, zinc finger
20	PeuTF04G00322.1	1	DONE	zf-Dof	Dof domain, zinc finger
21	PeuTF04G01143.1	1	DONE	zf-Dof	Dof domain, zinc finger
22	PeuTF17G00710.1	1	DONE	zf-Dof	Dof domain, zinc finger
23	PeuTF08G00503.1	1	DONE	zf-Dof	Dof domain, zinc finger
24	PeuTF11G00415.1	1	DONE	zf-Dof	Dof domain, zinc finger
25	PeuTF13G00671.1	1	DONE	zf-Dof	Dof domain, zinc finger
26	PeuTF12G00127.1	1	DONE	zf-Dof	Dof domain, zinc finger
27	PeuTF10G00899.1	1	DONE	zf-Dof	Dof domain, zinc finger
28	PeuTF03G01435.1	1	DONE	zf-Dof	Dof domain, zinc finger
29	PeuTF14G00964.1	1	DONE	zf-Dof	Dof domain, zinc finger
30	PeuTF02G01562.1	1	DONE	zf-Dof	Dof domain, zinc finger
31	PeuTF01G02355.1	1	DONE	zf-Dof	Dof domain, zinc finger
32	PeuTF07G00620.1	1	DONE	zf-Dof	Dof domain, zinc finger
33	PeuTF04G00502.1	1	DONE	zf-Dof	Dof domain, zinc finger
34	PeuTF05G01285.1	1	DONE	zf-Dof	Dof domain, zinc finger
35	PeuTF01G00832.1	1	DONE	zf-Dof	Dof domain, zinc finger
36	PeuTF15G00759.1	1	DONE	zf-Dof	Dof domain, zinc finger
37	PeuTF04G00383.1	1	DONE	zf-Dof	Dof domain, zinc finger
38	PeuTF08G00173.1	1	DONE	zf-Dof	Dof domain, zinc finger
39	PeuTF12G00722.1	1	DONE	zf-Dof	Dof domain, zinc finger
40	PeuTF02G01099.1	1	DONE	zf-Dof	Dof domain, zinc finger
41	PeuTF09G00304.1	1	DONE	zf-Dof	Dof domain, zinc finger
42	PeuTF10G00506.1	1	DONE	zf-Dof	Dof domain, zinc finger
43	PeuTF06G00823.1	1	DONE	zf-Dof	Dof domain, zinc finger

Figure S1. Confirmation of identified PeDofs using HMMER online software (https://www.ebi.ac.uk/Tools/hmmer/search/phmmer (accessed on 2 October 2024)). These 43 proteins all contain the Dof domain.

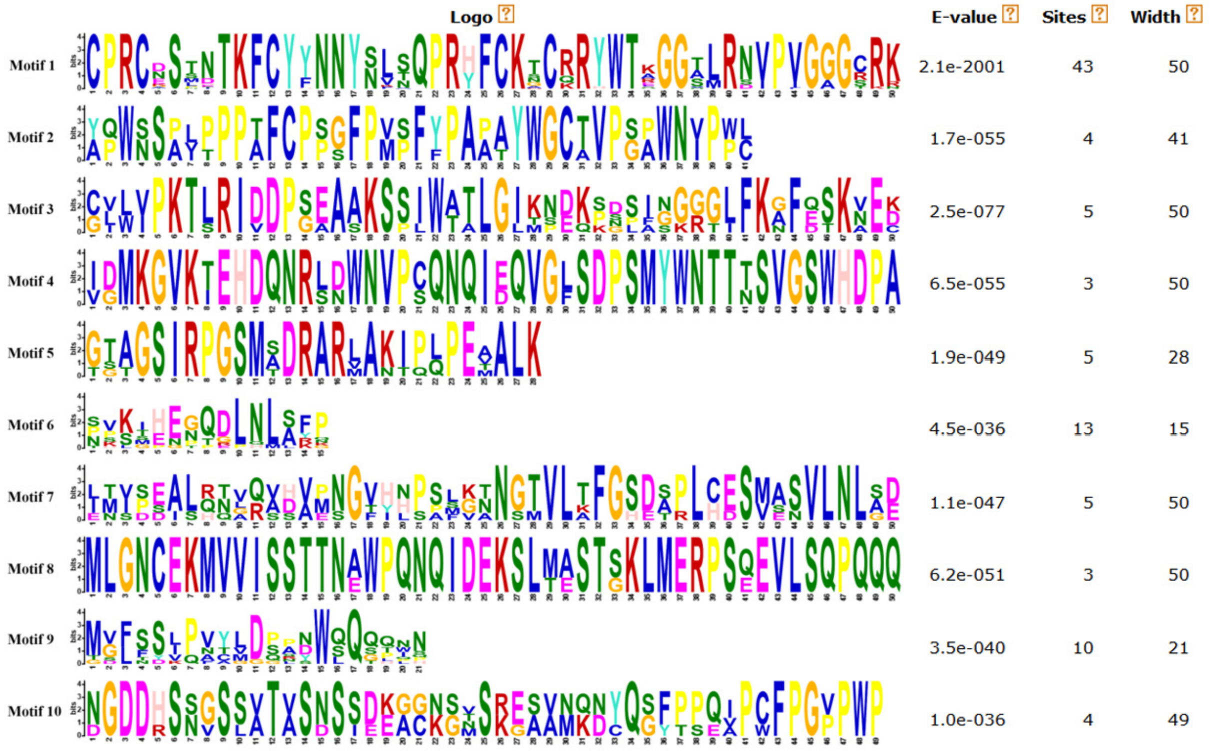

Figure S2. Prediction of conserved motifs in PeDof proteins. These 43 PeDof proteins contain 10 conserved motifs, which were predicted using MEME Suite (http://meme-suite.org (accessed on 2 October 2024)).

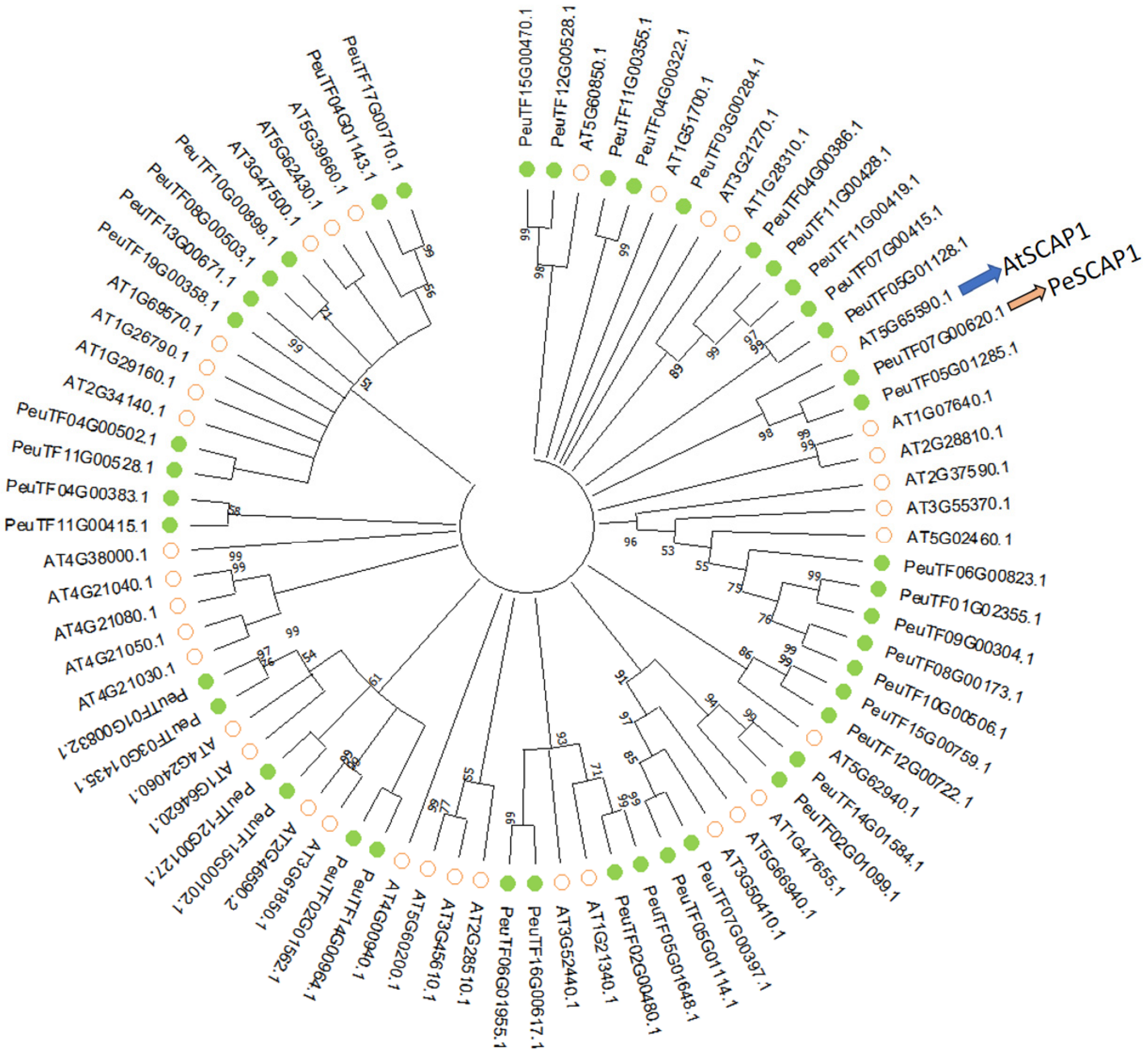

Figure S3. Neighbor-joining (NJ) tree of *P. euphratica* and *A. thaliana*. The bootstrap consensus tree inferred from 1000 replicates is taken to represent the evolutionary history of the taxa analyzed. Branches corresponding to partitions reproduced in less than 50% bootstrap replicates are collapsed. The evolutionary distances were computed using the JTT matrix-based method and are in the units of the number of amino acid substitutions per site. The rate variation among sites was modeled with a gamma distribution (shape parameter = 1). This analysis involved 79 amino acid sequences. There were a total of 618 positions in the final dataset. Evolutionary analyses were conducted in MEGA11. The blue arrow pointed to AtSCAP1, and the orange arrow pointed to PeSCAP1.

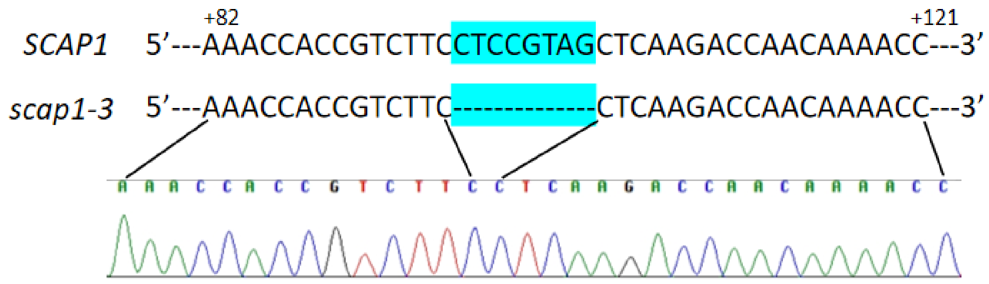

Figure S4. Sequencing analysis of *AtSCAP1* in the *atscap1-3* mutant. The *scap1-3* mutant, characterized by an 8 bp deletion in the *SCAP1* gene coding region, was derived from the T-DNA insertion mutant SALK_111683. This T-DNA is inserted into the *AT5G50850* (*MAB1*) gene, and the SALK_111683 mutant is actually the *mab1 scap1-3* double mutant. The *scap1-3* single mutant was isolated from F2 progeny of a cross between SALK_111683 and wild-type Col-0 plants.

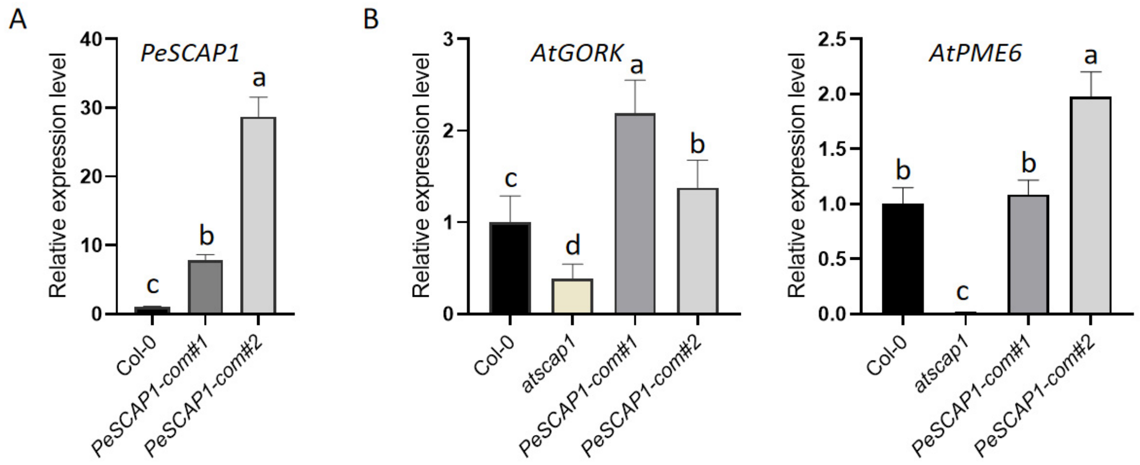

Figure S5. The relative expression levels of genes related to stomatal maturation. (A) Relative expression levels of *PeSCAP1* were compared among Col-0 and *PeSCAP1-com* lines. (B) Relative expression levels of *AtGORK* and *AtPME6* were compared among Col-0, *atscap1* and *PeSCAP1-com* lines. Total RNAs were extracted from the 7-day cotyledons. Values represented means ± SD (n = 9). Statistical analyses were conducted by One-way ANOVA with Tukey's test. Different letters above bars indicated statistically significant differences among genotypes (p<0.05).

Table S1 Primers used in this study.

Primer Name	Primer Sequence (5'-3')
**Complementation & overexpression**	
PeSCAP1-F	ACACGATCGATAAGCTTCCCATGGTGCCCCCAGATAATCTTC
PeSCAP1-R	TCGCCCTTGCTCACCATCCCGGGCAAAGCACTATACTGATGC
**Real-time qPCR**	
QPeSCAP1-F	CTCAAGTGACTGCAACGCCAAC
QPeSCAP1-R	GGGCAAAGCACTATACTGATGC
QAtGORK-F	gcatcagggaaacatgagtt
QAtGORK-R	gatacaagcagcagtgtgag
QAtPME6-F	gttataaagacggtgacagag
QAtPME6-R	ccgtagccgtaatccaatag
